# Supplementary material for: Covalent Organic Framework/Polyacrylonitrile Electrospun Nanofiber for Dispersive Solid-Phase Extraction of Trace Quinolones in Food Samples
Source: Nanomaterials (Basel). 2022 Jul 20;12(14):2482. doi: 10.3390/nano12142482 (PMC9319950; doi:10.3390/nano12142482)
Supplement: Supplementary file 1 [file nanomaterials-12-02482-s001.zip › nanomaterials-1807561-supplementary.pdf]

Supplementary Material

Covalent Organic Framework/Polyacrylonitrile Electrospun Nanofiber for Dispersive Solid-Phase Extraction of Trace Quinolones in Food Samples

Jinghui Zhou [1], An Chen [1], Hongying Guo [1], Yijun Li [1,2], Xiwen He [1], Langxing Chen [1,*] and Yukui Zhang [1,3]

[1] College of Chemistry, Tianjin Key Laboratory of Biosensing and Molecular Recognition, Nankai University, Tianjin 300071, China; 2120200750@mail.nankai.edu.cn (J.Z.); 1120180234@mail.nankai.edu.cn (A.C.); 1120190301@mail.nankai.edu.cn (H.G.); yijunli@nankai.edu.cn (Y.L.); xiwenhe@nankai.edu.cn (X.H.); ykzhang@dicp.ac.cn (Y.Z.)
[2] National Demonstration Center for Experimental Chemistry Education, Nankai University,
Tianjin 300071, China
[3] Dalian Institute of Chemical Physics, Chinese Academy of Sciences, Dalian 116023, China
* Correspondence: lxchen@nankai.edu.cn

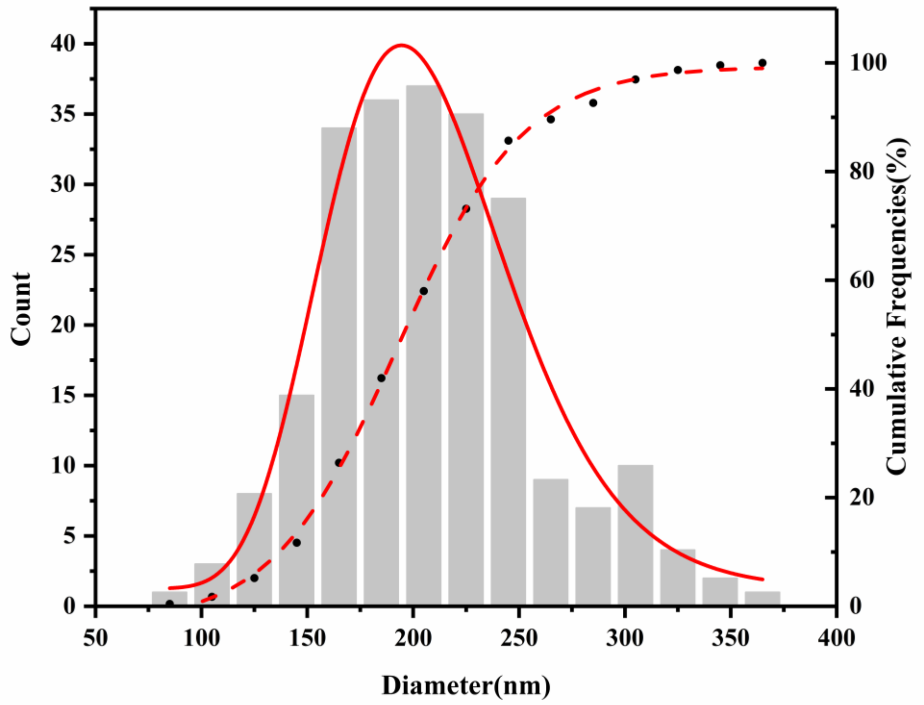

Figure S1. Size distribution and cumulative frequencies diagram of PAN nanofiber.

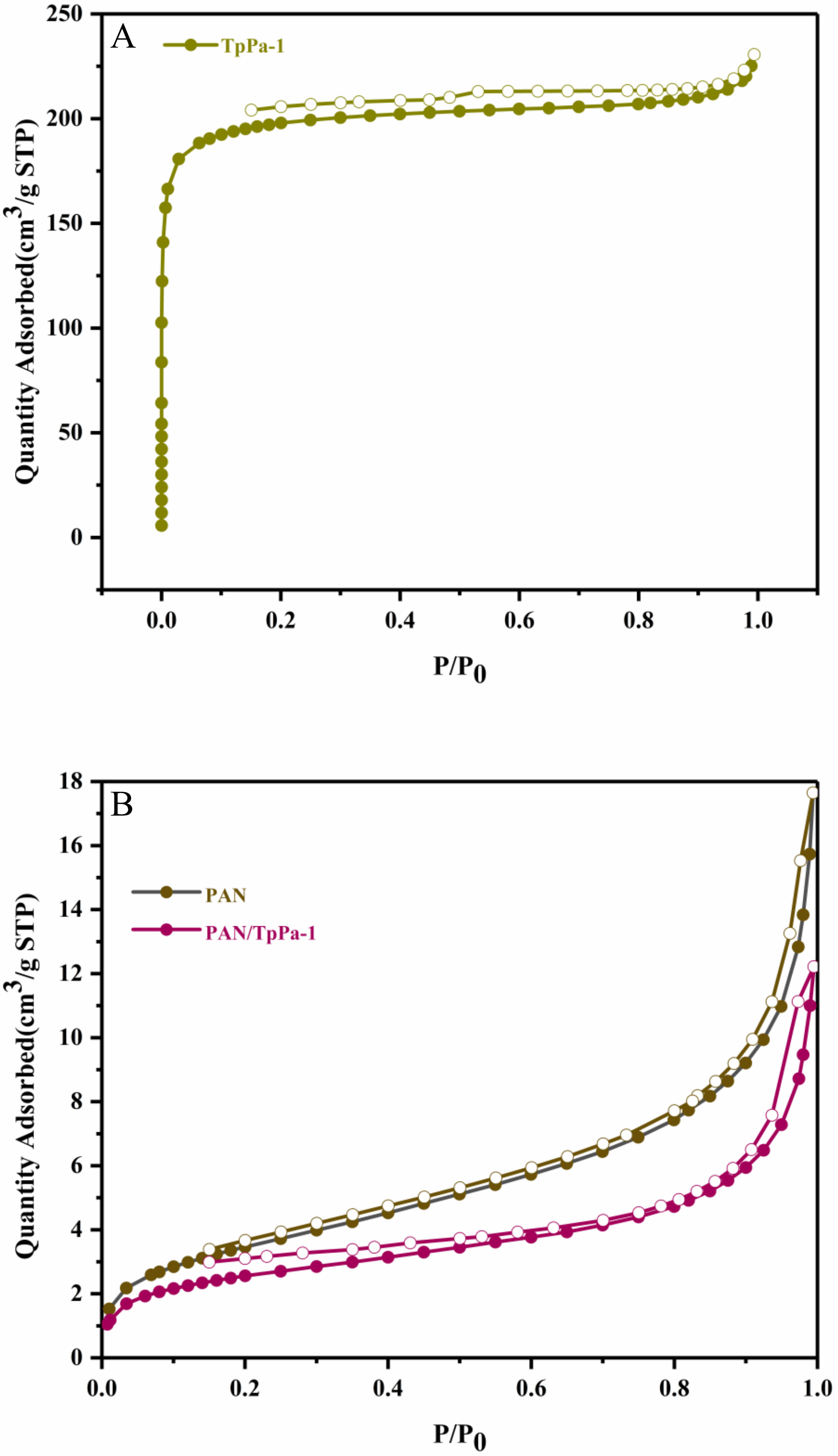

Figure S2. N_2 adsorption–desorption isotherm of TpPa–1 (A); PAN, PAN/TpPa–1 (B).

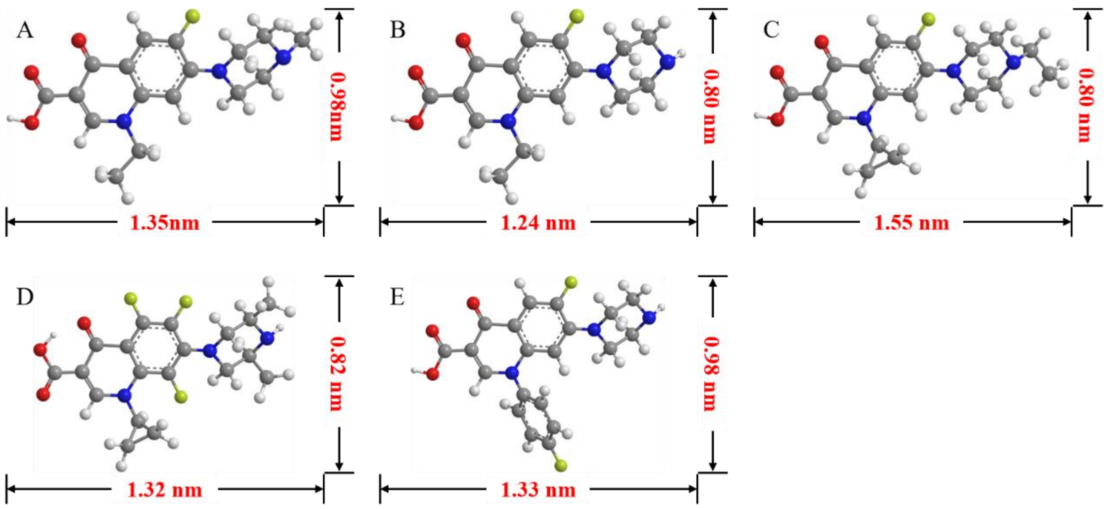

Figure S3. The molecular sizes of five quinolones PEF (A), NOR (B), ENR (C), ORB (D) and SAR (E) are illustrated by Gaussian simulation.

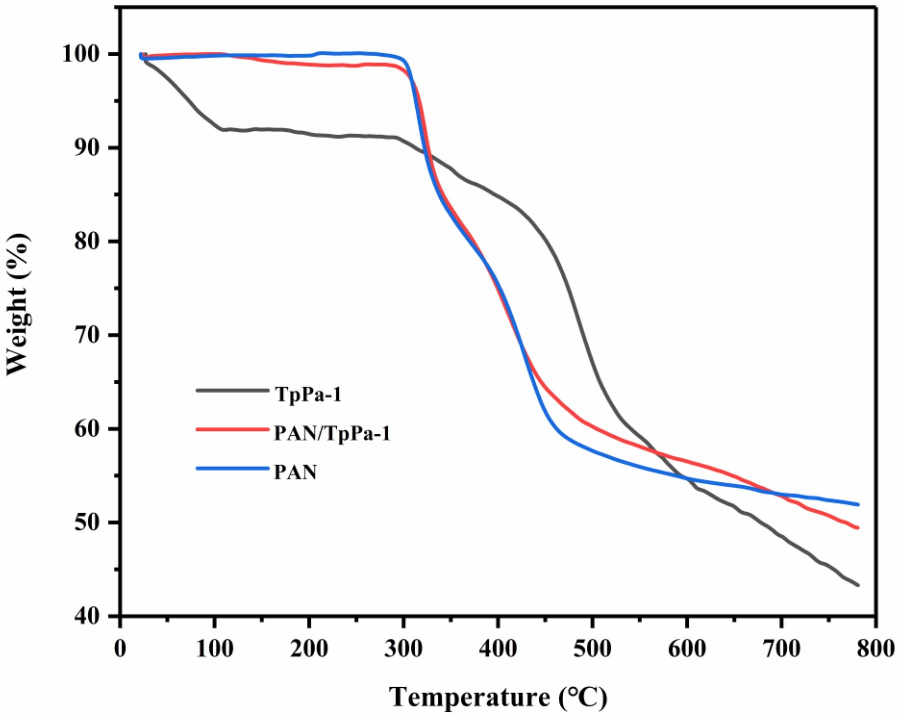

Figure S4. TGA results of TpPa–1, PAN and PAN/TpPa–1 electrospun nanofiber.

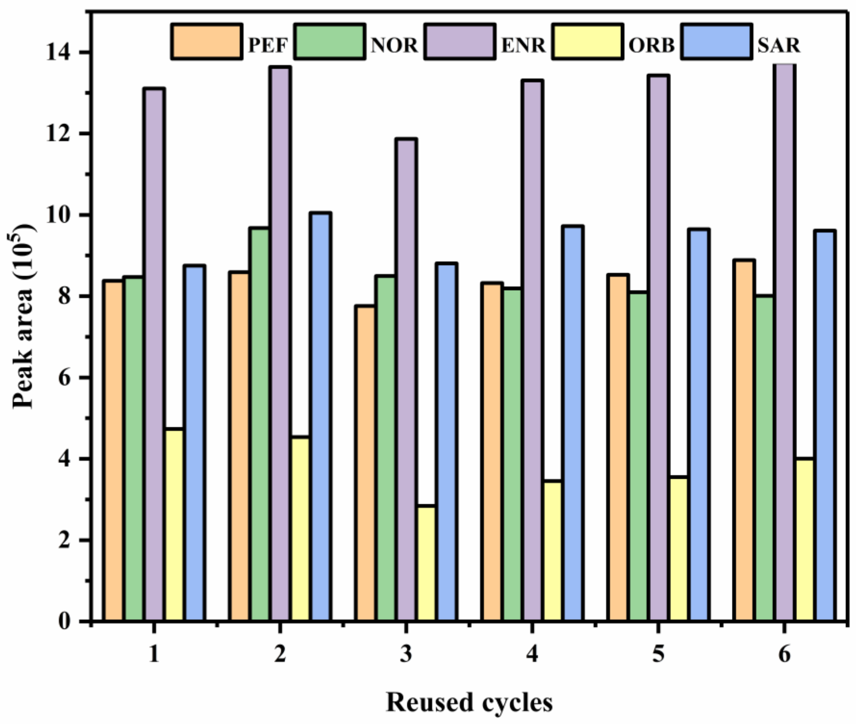

Figure S5. Reusability of PAN/TpPa–1 nanofiber.
